# Supplementary material for: Enhancing insights in sexually transmitted infection mapping: Syphilis in Forsyth County, North Carolina, a case study
Source: PLoS Comput Biol. 2024 Oct 31;20(10):e1012464. doi: 10.1371/journal.pcbi.1012464 (PMC11774488; doi:10.1371/journal.pcbi.1012464)
Supplement: Attachment — Submitted filename: responseToEditor.docx [file pcbi.1012464.s001.docx]

Thank you for your detailed comments and suggestions for improvements to our manuscript. Your comments have enhanced our article and we have put substantial effort into addressing your thoughtful points raised during the review process. The article has been comprehensively rewritten to make our goals clearer, defend the use of the data and to remove jargon to make the paper easier to comprehend. We have gone through each of the reviewers and editors comments and revised the article accordingly. This letter details the edits we have made to revise our manuscript. Points raised are noted in blue and our responses are in black.  

Editor Comments

1. However, I share the reviewer concerns about whether data from almost 15 years ago is still relevant for understanding current trends. My advice to the authors is that a strong focus of the revision needs to be on defending the use of this time period. If more recent data are not available for the study area, perhaps adding some plots about state- and national-trends to help contextualize where things were in 2008-11 vs today?
   a. The introduction was completely revised and new paragraphs were added to address the continued importance of this data even though it is from 2010s. Lines 83-119 of the introduction outline the importance of this dataset. Plot added (new Figure 1) to the article along with additional contextualizing text. Additionally in the discussion we add new paragraphs to contextualize our results to current issues in syphilis, lines 702-740 and 755-764. Additionally 25 new references and information from them were added to better relate this research to current issues with syphilis, STIs, partnership selection.
2. In addition, I also agree that the authors should take care during the revision to clarify the specifics of what was done, assumed in the model, and found via analysis of the model.
   a. The introduction, methods and results sections were heavily revised to increase a reader’s ability to understand our modelling process and its results.
3. Particular focus should be placed on linking terminology/approaches from geospatial ecology to infectious disease epidemiology. As the authors understand, conceptually the approaches are quite similar, but the jargon will be a barrier to an interdisciplinary read/understanding of this work.
   a. Jargon was removed and extensive edits were made to better contextualize the research.
4. Finally, please ensure you share the code the analysis in compliance with our code-sharing policy.
   a. Code is being uploaded to our website to be freely share. This paragraph was also added to the text.

“The BME numerical processing was performed using MATLAB 7.8 [70] and the BMElib version 2.0c [71]. The code created and used in the analysis can be found at: <https://mserre.sph.unc.edu/BMElab_web/mappingStudies/Syp_ForsythNC/>. The BMElib code is freely available at: <https://mserre.sph.unc.edu/BMElib_web/> . For those who do not have programming knowledge there is also a graphical user interface version of BME (BMEGUI) that guides a user through the BME process, download it at: <https://mserre.sph.unc.edu/BMElab_web/> [19].”

Reviewer #1:

1. the submission lacks some clarity in its stated goals.
   a. Abstract has been revised to have a better definition of our goals and a clearer description of our goals in in the introduction, lines 111-115 and 194-206.
2. It was not clear how the analysis demonstrated that the moving window and UMBME was the better approach.
   a. The methods were rewritten to better explain our analytical methodology to compare the results using latent rate theory and the Percent Change in Mean Square Error (Lines 454-502). The results then were re-organized and re-written to better explain the method cross validation results (Lines 507-524).
3. Additionally, the data used are more than a decade old which may have limited the utility of the analysis.
   a. The usefulness of the dataset is now thoroughly explained in the article. Multiple paragraphs have been added to the introduction along with a plot to address the continued importance of this data even though it is from 2010s (Lines 83-119). Furthermore, the discussion now more clearly discusses and relates the results to current issues in syphilis, lines 702-740 and 755-764.
4. Syphilis rates have increased broadly since 2011 and the epidemic is much less MSM focused.
   a. This article has been added to the discussion to address this, “Some of the dynamics of syphilis transmission have changed since 2008. First, in the US overall the rate of primary and secondary syphilis for men who have sex with men (MSM) has decreased from 72% in 2010 [74] to 45% in MSM with an additional 20% in men who have sex with unknown partners (MSU) in 2021 [2]. However, some of the 2021 MSU data likely could be categorized within MSM [75-76]. Conversely, in North Carolina in 2022 the rate for gay, bisexual and other men who have sex with men was estimated to be 1,054 per 100, 000 whereas the rates of men who only have sex with women and the overall rate for women were both 17 per 100,000 [29]. Thus in North Carolina the primary high risk population may not have significantly changed. Additionally may of the rates seen in the 2008-2011 Forsyth outbreak are similar to 2021 syphilis rates in the North Carolina, where the county rates in the 20 most affected counties ranged from 54.8-23.5 [14]. The gender change in syphilis transmission in the US in general from 2011 to 2021 could affect the application of the results of geographical transmission shown in this study to outbreaks outside of North Carolina.”
5. Additionally, the wide-spread adoption of online and cell-phone based apps for meeting partners has changed the community level risk space for syphilis. As a result, some of the assumptions around residential geography may not be the same as in 2008-2011. For example, fewer people may meet partners at physical community sites (e.g., bars, parks, schools) and rely more on app based approaches – this would alter the way geography may play into syphilis risk.

New references and the following paragraph has been added to the discussion addressing this, “Additionally methods for partnership selection have changed substantially since 2011. In 2008-2011 people often “cruised”, i.e. meeting partners at physical environments (e.g., bathhouses, parks, parks, pools, malls). With the wide-spread adoption of internet based phones, most people now rely on apps and online dating websites for partner selection changing the community level risk space for syphilis [73; 77-80]. However studies have also shown participants who have a more challenging time with dating apps, still frequent common activity spaces to meet partners [73; 80]. Yet even in the dating app era, localized geographic proximity is still a key factor partner selection for both men and women [73; 80-81]. In practice, distance travelled to meet sexual partners may not have drastically changed over time. Interviews on partner selection have shown participants preferentially selected partners for whom they had to travel the shortest distance possible to connect [73; 80]. Research continues to support that partnership selection is a localized event where this selection occurs online or offline [73; 80-83]. Therefore many of the assumptions around geographical partner selection dynamics may remain in 2024.”

6. Additionally, since the data was collected, the underlying population may have changed geographically in important ways. For example, has the distribution of MSM changed in the geographic area? This would alter the findings and modify the spatial risk, which may result in different findings.

This is an interesting point, but outside of the scope of the current analysis. We did add this paragraph to the analysis to contextualize how our results fit into the current context of syphilis transmission.

“Use of our approach may benefit health authorities by providing greater resolution of ongoing outbreaks, allowing for targeted intervention and resource allocation [12; 19; 27; 44; 65; 73]. Even if the spatial dynamics of STI transmission have changed it is still important to have highly accurate visual representations STI data. Understanding the spatial progression of outbreaks can help to contextualize current and past outbreaks. Understanding the geographic progression of an STI outbreak can help describe the spread within and outside sexual core transmitters and their sexual network [73]. These insights can provide public health officials a clearer view of the sexual network location and their potential activity spaces (where the sexual behaviors are actually occurring) [12; 19-20; 27; 73] to isolate areas of need and inform intervention strategy selection and development [73]. Our analysis showed the syphilis outbreak hotspots were highly localized, concentrated to small areas, but persist for a relatively long time, giving a better idea where to target resources.”

7. While I understand more contemporary data may not be available, I encourage the authors to explain more about: (1) why the older data were used, (2) the reasons why more recent data may not be available, and (3) what are the potential implications in translating their findings data collected more recently.
   a. Thank you, we are addressing this issue throughout the article- in both the introduction and the discussion.
8. -Similar analyses to others presented by this group. I do however wonder what this analysis adds to the literature beyond their prior work. A similar analysis has been recently published in PLOS Global Health and other papers have been published over the past decade. I encourage the authors to address how this analysis is different and novel and what it adds to the literature beyond their prior stellar work.
   a. We have thoroughly revised the introduction and address our past study including lines, 194-198, “Past STI studies by Fox et al [19] found the edge effect is especially prevalent in the prediction method, kriging where rates are highest at the prediction point. This phenomenon is known as “kriging islands”. Kriging islands can make maps more difficult to interpret by obscuring the ability to fully understand the progression of an outbreak and how it moves between susceptible communities [19]. “ Furthermore, we address that little research exists on outbreaks in North Carolina since 2011, and syphilis outbreaks in the US. Even less research exists on geographic information on syphilis outbreaks and this research fills in that gap, lines 83-115.
9. Improved clarity in analytic goals and results: From my reading, the goal of this analysis was to examine different spatial methods to reduce bias from ecological fallacy and edge effects. This was not super clear and took some digging around to figure this out. The way the results are organized, this goal is cursorily addressed and not very clearly. I recommend the authors review and make clear the goals and how the results meet these goals.
   a. The results have been comprehensively re-organized to make the goals and how they were met clearer to interpret.
10. Background – some important concepts are not very well described. For example, lines 83-5 these should be explained more clearly to an audience who may not have a depth of knowledge in spatial work.
    a. This section was completely re-written to make more sense and have less jargon. “Centroid assignment to geopolitical boundaries can obscure pertinent geographical information needed to conduct effective disease mapping [19; 37] and cause multiple geostatistical issues. These include the: 1) Modifiable Areal Unit Problem (MAUP); 2) edge effect [19; 38-39]; 3) kriging islands of artificially higher and lower incidence at centroids [19]; 4) potentially masked hotspots (hotspots are localized geographical areas with elevated incidence rates) [19; 37]; 5) a background rate greater than zero; and 6) the small number problem [19]. “
11. -Lines 94-5 what is meant by “connectivity” and “geographical extent”?

These lines have been re-written for clarity and now read, “The edge effect results from displacing an incidence rate to a geographical centroid causing ecological bias. In a map it is shown as hotspots not located in the correct geographic location, the complete loss of a hotspot and/or multiple hotspots incorrectly connected or disconnected to each other”

12. -Line 145- why was the CDC IRB review conducted?
    a. Text edited for clarity it now reads “This study was reviewed by the University of North Carolina Committee on the Protection of the Rights of Human Subjects and was determined to be exempt, based on exemption criterion 4 (IRB 05-3080).”
13. -Lines 150-7- I did not follow the three geographic indicators and how they were used to plot one location?

The section on the geographic locators was revised to flow better and be more easily understandable. It now reads, “The first geocoding round used our highest quality and primary locator created by the North Carolina Department of Transportation containing street-level geographical data. Cases not matched using our first locator were then matched using our secondary locator created by the North Carolina Emergency Response System which contains GPS point locations for households. Cases not matched to the first or second locator were then matched to our tertiary locator created using ESRI’s 2006 Street Map shapefile [59], which is primarily used for locating residences with outdated street names, prisons, and military bases. Finally, cases with a post office box address were spatially assigned to that post office address. Matching in this method allows for the highest quality geographic data to be linked to the cases while maximizing the number of cases matched to a geolocator.”

14. -Lines 158-66 – were comparisons made between early syphilis cases that could and could not be geocoded? Any important differences?
    a. Addressed- and this information was added- “The data that could not be geocoded appeared to be missing at random without any noticeable patterns. We believe the missing data does not have a consequential on the final results and only adds noise rather than an inherent bias.”
15. Line 174- Is ref 44 correct here?
    a. Thank you, this was a mistake that has now been corrected, a reference was accidentally left off the list and now has been added and corrected. Schumacher C, Bernstein K, Zenilman J, Rompalo A. Reassessing a large-scale syphilis epidemic using an estimated infection date. Sexually transmitted diseases. 2005 Nov 1:659–64. pmid:16254539.
16. Lines 177-92- I found this section quite unclear. Figure 1 is not great resolution and I did not understand what was solid turquoise circle.

Figure was recreated with better resolution and uploaded. Text is also edited for clarity and now reads as follows” Two datasets of syphilis incidence rates were created and compared, US census block groups (CBG) and uniform grid with a moving window (Figure 2). Our first dataset, composed of the traditional, geopolitical boundaries was created in ESRI’s ArcGIS 10 [58] using the US 2000 Census populations and census block group boundaries packaged shapefile [58]. This file was used to calculate the CBG centroids. Figure 2 clearly shows the irregular locations of the CBG centroids, size and shape demonstrating a strong potential for MAUP and the edge effect. We hypothesize kriging islands, MAUP and edge effect created when aggregating to geopolitical incidence groups can be corrected by creating a uniform grid with a moving window of overlapping perfect circles (shown as turquoise dots with blue circles in Figure 2). The uniform grid approach changes the area of aggregation from a geopolitical boundary to a standardized area with a consistent centroid location allowing aggregation without the variability caused by size and shape [38-40]. Furthermore, overlapping the boundaries of the uniform grid area creates a contiunuous surface, reducing the edge effect [39-41]. Cases located within each of these grid-based incidence areas are identified and the underlying population within the ubiquitous area is systematically calculated and used to construct a rate assigned to each grid centroid.”

17. Lines 200-252- I also did not follow how denominator data was obtained for the circle approach. CBG have known populations to use as denominators, how were these used to estimate the incidence rates for these area? Doesn’t the approach used basically imply the same issues as edge effects and ecologic fallacy? It assumes a uniform population within a CBG which is not likely, so how does this approach help address the issues brought up in the intro?

This section was completely revised and the uniform population issues and edge effect are discussed. It now reads, “To calculate incidence rates, CBG cases were aggregated spatially by census block group boundaries, and temporally with a rolling period of 6 months duration to lessen the small number problem. Population growth was also incorporated into the crude rate calculation through a linear interpolation of the census block group population for all 12-64-year-olds in 2000 and 2007 assuming positive population growth over the time period. The crude incidence is denoted as *R_ij_* and calculated as *R_ij_=y_ij_/*(*n_ij_T*). Incidence period of duration *T* expressed in years (i.e. *T* = 0.5yr for a 6 month incidence), centered at time *t_j_*, *j*=1 denote the number of rolling time periods (141 rolling 6-month aggregated time periods with monthly estimates). *i*=1 denotes the incidence area (5259 census block group centroids, 10376 ubiquitous group centroids), *y_ij_* is the number of incident syphilis cases and *n_ij_* is the population at time *t_j_*. Time periods that did not contain incident syphilis reports were assumed to have a rate of 0 cases per 10,000. The CBG dataset is composed of these crude rates.

To construct rates for the uniform grid based method, the CBG *R_ij_* rates are enriched with a grid-based incidence rate field. The uniform grid is a series of grid points constructed solely within Forsyth County. The distance between grid points, *D* was set using the following equation: $D=f*\frac{1}{n}\left( \sum_{a=1}^{n} \frac{\sqrt{A_{a}}}{\pi} \right)$, where *n* is the user selected number of CBGs (we used 5), *A_a_* is the area of the a*^th^* CBG (i.e. 1 of 5), $\frac{1}{n}\left( \sum_{a=1}^{n} \frac{\sqrt{A_{a}}}{\pi} \right)$ calculates the average CBG area, and *f* is the factor used to increase or reduce the spatial resolution of the grid. For this study we used *f* = 0.65 to create a fine grid lattice of grid points throughout the study area.

Each uniform grid (UG) boundaries is a perfect circle with a radius *r_i_*, where *i* is the spatial location of the uniform grid centroid/point. *r_i_* is calculated by using an inverse weighted distance area average of the five closest CBGs to *r_i_*. It is shown as, $r_{i}=\sum_{a=1}^{5} d_{ia}^{-1}*\frac{\sqrt{A_{a}}}{\pi}/\sum_{a=1}^{5} d_{ia}^{-1}$ where *d_ia_* is the distance between the grid point and its *a^th^* CBG neighbor. This approach allows for a gradual change in the size of the uniform grid areas in relation to their local CBGs, as seen in Figure 2. The boundaries of the uniform grid overlap smoothing the rate field and reducing the edge effect.

The population of the uniform incidence area is a summation of the proportion of populations from each census block groups it overlaps. Calculated as: $n_{ij}=\sum_{a=1}^{n} \frac{\left\| \mathrm{CBG}_{a} \cap\mathrm{UG}_{i} \right\|}{\left\| \mathrm{CBG}_{a} \right\|}n_{aj}$. Where the percentage of every census block group within the uniform grid incidence area was calculated, where$\left\| \mathrm{AG}_{a}\cap\mathrm{UG}_{i} \right\|$ is the area of the intersection between CBG *a* and uniform grid (UG) boundary *I and* is the proportion of the area of *CBG_a_* in the uniform grid (UG) boundary *i*. This value is then multiplied by *n_aj_*, the CBG population in *CBG_a_* at time *t_j_*. $\frac{\left\| \mathrm{AG}_{a}\cap\mathrm{UG}_{i} \right\|}{\left\| \mathrm{AG}_{a} \right\|}$These population values were summed to calculate the total population for the uniform grid incidence area. This approach assumes a uniform population distribution within the CBG. The uniformity within the grid centroid placement pulls the populations from multiple centroids, creating a modeled population at each point to smooth some of the concerns with population homogeneity assumption.

To continue to increase spatial accuracy of the dataset, we reverse calculated the donut geomask for each case. The number of cases within each uniform incidence area was calculated as a function of the probability that a geomasked case is within the uniform grid. The following is known about the geomasked cases: 1) each case is geomasked within the AG they are located in and 2) there is a maximum and minimum distance a case can be moved from its original location [28; 44-45] (these values can only be accessed on secure computers onsite at the NC- DHHS by selected researchers). From this we can infer that the probability $w_{li}$ of the^th^ geomasked case at time $t_{j}$ in area is $w_{\mathrm{li}}\approx\frac{\left\| \mathrm{RDAG}_{l}\cap\mathrm{UG}_{i} \right\|}{\left\| \mathrm{RDAG}_{l} \right\|}$. *RDCBG_l_*$\mathrm{RDAG}_{l}$ is geographic area of original donut area case *l* was initially located in its CBG before geomasking. More specifically,$\mathrm{RDAG}_{l}=\mathrm{RD}_{l}\cap\mathrm{AG}_{l}$, where *RD_l_* is the reverse geomasked donut centered on the geomasked location of case *l*, and *CBG_l_* $\mathrm{AG}_{l}$ is the Census Block Group area that case *l* is located in. Then, the uniform grid incidence rate over area at time $t_{j}$ is calculated as $R_{ij}\approx\frac{\sum_{l=1}^{N_{j}} w_{li}}{n_{ij}T}$, where $N_{j}$ is the total number of cases at time $t_{j}$, and $\sum_{l=1}^{N_{j}} w_{li}$ is the sum of the probabilities that each case at time $t_{j}$ is located in ${UG}_{i}$. “

18. Lines 329- The text reads Aug 2008-Mar 2010, but other places (like the abstract) say data were used from 2008-2011. This needs to be clarified and consistent throughout.
    a. This was changed throughout and the dates are stated 2008-2011.
19. Line380-1: The statement about endemic syphilis being <=15 cases/100,000 needs a reference. I have never heard this before.
    a. Reference added and text edited. Hook EW. Is elimination of endemic syphilis transmission a realistic goal for the USA?. The Lancet. 1998 Jun 1;351:S19-21. The text now reads, “By February-July, 2011 the Winston-Salem incidence has returned to endemic levels of 3-7 cases per 100,000 and the outbreak has subsided. Endemic is when the incidence is low and stable, and was defined in a study by Hook in the Lancet in 1998 as an area with 4 or more syphilis cases per 100,000 [72]. Furthermore, the Kernersville and Lewisville syphilis rate returns to 1-5 cases 10,000person-years. Figure 6 and the movie display the progression of the syphilis epidemic across Forsyth County between 2008 –2010 with its three largest towns labeled (Winston-Salem, Lewisville, and Kernersville). “
20. Figure 1: the labels in the text don’t match the figure, what is ubiquitous and arbitrary mean here?
    a. Figures 4 & 5 were edited to remove this verbiage and increase clarity
21. Line 424-426: I don’t think the results presented show this.

The methods and results section was rewritten to better address how our method mathematically shows increased accuracy in lines 453-503 to better describe our method cross-validation analysis. Furthermore, former lines 424-426 were also edited and now read, “We show combining a moving window and a UMBME analysis with geomasked data produced more realistic geographical patterns for more accurate and targeted insights into a syphilis outbreak. The improved visualization in the maps was accompanied by less apparent error, with the uniform grid model performing 5-26% better in predicting the true rate compared to the traditional, geopolitical CBG based model.“

22. Lines 438-440- data presented don’t show this.

These lines have now been clarified to read as, “Our approach is particularly novel because we introduce the ability to remove what is commonly referred to as “kriging islands”, where rates are higher and lower at centroids [19], providing greater sensitivity for hotspot detection. This enhanced visualization is especially important when hotspots are located on or across the boundary of two or more aggregation areas which traditionally can result in the complete loss of a hotspot. Furthermore, removing kriging islands reduces the background rate which unmasks rural hotspot areas of concern. Removing kriging islands resulted in an increased ability to visualize spatial corridors of incidence and a more accurate representation of interconnection and spread.”

Reviewer #2:

1. The dates of cases included in the study are unclear to me as different date ranges are described at different points in the paper.
   a. The dates are 2008-2011 and it has been fixed and standardized throughout the article.
2. While this is appropriate when describing the rolling windows in the results and the date range of the specific outbreak, in other locations in the manuscript it raises confusion: eg. In the methods, are all syphilis diagnoses between 1999-2011 included or is it 2008-2011 as is stated elsewhere. Suggest clarifying the differing date ranges listed throughout the manuscript
   a. We removed the line referring to 1999-2011 because only data between 2008-2011 were utilized for this study.
3. Suggest either limiting to P&S syphilis cases or describing potential limitations of including early latent syphilis cases. While this is unlikely to be an issue for this study as it focuses on a single county, the formal case definition of early latent syphilis was known to cause confusion when staging the case, leading to potential misstaging and issues with generalizing between locales, so generally, reporting on syphilis surveillance is restricted to P&S cases. Additionally, this stage has since been revised to early non-primary non-secondary (ENPNS) syphilis with an updated case definition
   a. These lines were added to the article, “As of 2023 the NC DHHS studies and utilizes the category “early syphilis” [14]. To support the work of the DHHS who both provided the data for the project and supported it, the early syphilis categories (primary, secondary and early latent) were also used in our analysis. Early syphilis is the stages when the syphilis pathogen has highest infectivity for sexual partner transmission. Late latent syphilis is not considered infectious and excluded from the analysis [20; 44].”
4. *Additional descriptives could help with the interpretation of later results. For example, a table detailing # of cases by stage and/or year and/or # of excluded cases by stage/year*

This would be an interesting table, but we are unable to create this table in a reasonable time period because of data protection issues.

5. Back-dating to estimate syphilis transmission dates: I’m unfamiliar with these specific date ranges and the provided citation- (44. Choi KM, Serre ML, Christakos G. Efficient mapping of California mortality fields at different spatial scales. Journal of Exposure Science & Environmental Epidemiology. 2003 Mar;13(2):120-33) doesn’t seem to mention syphilis
   a. Thank you, this was a mistake that has now been corrected, a reference was accidentally left off the list and now has been added and corrected. Schumacher C, Bernstein K, Zenilman J, Rompalo A. Reassessing a large-scale syphilis epidemic using an estimated infection date. Sexually transmitted diseases. 2005 Nov 1:659–64. pmid:16254539.
6. Provide a citation for rate definition of endemic syphilis (lines 379-381)
   a. This has been fixed, reference added and was pointed out by another reviewer. (New reference, #72: Hook EW. Is elimination of endemic syphilis transmission a realistic goal for the USA?. The Lancet. 1998 Jun 1;351:S19-21.)
7. I would be interested in some clarification of the implications of the finding that many hotspots seem to be located near popular meeting locations (lines 438-440 and 455-457).

This paragraph has been added to the discussion contextualizing hotspots located near popular meeting spots to current issues in partnership selection.” Additionally methods for partnership selection have changed substantially since 2011. In 2008-2011 people often “cruised”, i.e. meeting partners at physical environments (e.g., bathhouses, parks, parks, pools, malls). With the wide-spread adoption of internet based phones, most people now rely on apps and online dating websites for partner selection changing the community level risk space for syphilis [73; 77-80]. However studies have also shown participants who have a more challenging time with dating apps, still frequent common activity spaces to meet partners [73; 80]. Yet even in the dating app era, localized geographic proximity is still a key factor partner selection for both men and women [73; 80-81]. In practice, distance travelled to meet sexual partners may not have drastically changed over time. Interviews on partner selection have shown participants preferentially selected partners for whom they had to travel the shortest distance possible to connect [73; 80]. Research continues to support that partnership selection is a localized event where this selection occurs online or offline [73; 80-83]. Therefore many of the assumptions around geographical partner selection dynamics may remain in 2024.”

This line was also added to the discussion, “These insights can provide public health officials a clearer view of the sexual network location and their potential activity spaces (where the sexual behaviors are actually occurring) [12; 19-20; 27; 73] to isolate areas of need and inform intervention strategy selection and development [73].”

Reviewer #3:

1. Present references for choosing the quantities described between lines 218 and 223. In particular: when D=0.5 is chosen, this is the value of the equation presented in line 219, but the average CBG radius is not defined?

We agree that this section was not clearly written, thus it was rewritten to read as, “	To construct rates for the uniform grid based method, the CBG *R_ij_* rates are enriched with a grid-based incidence rate field. The uniform grid is a series of grid points constructed solely within Forsyth County. The distance between grid points, *D* was set using the following equation: $D=f*\frac{1}{n}\left( \sum_{a=1}^{n} \frac{\sqrt{A_{a}}}{\pi} \right)$, where *n* is the user selected number of CBGs (we used 5), *A_a_* is the area of the a*^th^* CBG (i.e. 1 of 5), $\frac{1}{n}\left( \sum_{a=1}^{n} \frac{\sqrt{A_{a}}}{\pi} \right)$ calculates the average CBG area, and *f* is the factor used to increase or reduce the spatial resolution of the grid. For this study we used *f* = 0.65 to create a fine grid lattice of grid points throughout the study area.”

2. The word radii in line 226 was not clear to me.

We agree that this section was not clear, thus it was revised. It now reads, “Each uniform grid (UG) boundaries is a perfect circle with a radius *r_i_*, where *i* is the spatial location of the uniform grid centroid/point. *r_i_* is calculated by using an inverse weighted distance area average of the five closest CBGs to *r_i_*. It is shown as, $r_{i}=\sum_{a=1}^{5} d_{ia}^{-1}*\frac{\sqrt{A_{a}}}{\pi}/\sum_{a=1}^{5} d_{ia}^{-1}$ where *d_ia_* is the distance between the grid point and its *a^th^* CBG neighbor.”

3. Present references for how calculate the number of cases within each uniform incidence area.

The methods presented in this article are novel; therefore there were not references that this was based upon. The rate is the standard incidence rate calculation of the number of cases within a chosen area.

4. The quantity Wli, defined in line 245, needs to be better explained.

We agree this section needed to be revised. It now reads, “To continue to increase spatial accuracy of the dataset, we reverse calculated the donut geomask for each case. The number of cases within each uniform incidence area was calculated as a function of the probability that a geomasked case is within the uniform grid. The following is known about the geomasked cases: 1) each case is geomasked within the AG they are located in and 2) there is a maximum and minimum distance a case can be moved from its original location [28; 44-45] (these values can only be accessed on secure computers onsite at the NC- DHHS by selected researchers). From this we can infer that the probability $w_{li}$ of the^th^ geomasked case at time $t_{j}$ in area is $w_{\mathrm{li}}\approx\frac{\left\| \mathrm{RDAG}_{l}\cap\mathrm{UG}_{i} \right\|}{\left\| \mathrm{RDAG}_{l} \right\|}$. *RDCBG_l_*$\mathrm{RDAG}_{l}$ is geographic area of original donut area case *l* was initially located in its CBG before geomasking.

5. How to calculate Reverse Donut area? Wouldn't it be more appropriate to count the number of cases in each UG, since the occurrences are already geomasked?
   a. That is definitely a good point, however we were attempting to estimate what the original location of the points were prior to aggregating to both protect patient privacy and increase spatial accuracy. Thus, we added this line to address this issue, “To continue to increase spatial accuracy of the dataset, we reverse calculated the donut geomask for each case.”
